# Dimensional stability of short fibre reinforced flowable dental composites

**DOI:** 10.1038/s41598-021-83947-x

**Published:** 2021-02-25

**Authors:** Raju Raju, Ginu Rajan, Paul Farrar, B. Gangadhara Prusty

**Affiliations:** 1 School of Mechanical and Manufacturing Engineering, University of New South Wales, Sydney, NSW 2052 Australia; 2 School of Electrical, Computer & Telecommunications Engineering, University of Wollongong, Wollongong, NSW 2522 Australia; 3 SDI Limited, Melbourne, VIC 3153 Australia; 4 ARC Centre for Automated Manufacture of Advanced Composites, School of Mechanical and Manufacturing Engineering, University of New South Wales, Sydney, NSW 2052 Australia

**Keywords:** Engineering, Materials science

## Abstract

Fibre-reinforced dental composites are proven to have superior mechanical properties in comparison with micro/nano/hybrid filled composites. However, the addition of small quantities of short glass fibres could affect the dimensional stability of the restoration both during initial stages as well as through the life of the restoration. This in-vitro study aims at evaluating the physical properties of short S-Glass reinforced flowable dental composites. Two S-Glass short fibre-particulate reinforced (5 wt% of aspect ratios 50 and 70) and one particulate only reinforced flowable dental composites were prepared with UDMA-TEGDMA based dental monomer systems. Samples were photopolymersied for 60 s and stored in distilled water at 37 °C for 24 h before testing. Depth of cure (through-thickness microhardness), volumetric shrinkage (Archimedes technique), polymerisation stress (cantilever based tensometer), curing exotherm (thermocouple), water sorption and solubility (ISO 4049) and thermal expansion coefficient (dilatometer) were determined. The test results were statistically analysed using one-way ANOVA (p < 0.05). Depth of cure increased by 41%, volumetric shrinkage increased by 8.3%, shrinkage stress increased by 37.6%, exotherm increased by 20.2%, and thermal expansion reduced by 6.4% while water sorption and solubility had a negligible effect with the inclusion of short glass fibres. The study demonstrates that within the same organic resin system and quantity, a small replacement of fillers with short fibres could significantly affect the dimensional stability of the composite system. In conjunction with mechanical properties, this study could help clinicians to gain confidence in fibre reinforced dental composite restorative system.

## Introduction

The dental restorative industry has been witnessing a radical transformation in recent years, moving away from the traditional approach towards new alternative materials, providing better results^1^. Due to their ease of handling and ability to penetrate in the intricate spaces of cavities, flowable composites are favoured in both conventional and bulk-fill modes. Flowable composites also enable nozzle extrusion, reduced working time and lower costs^2^. However, studies have shown that the resin-based composites are inferior to amalgam with comparable mechanical, physical properties, and survival rates. Reduced mechanical and physical performance of the dental composite are attributed towards its high strength inorganic fillers in a weak organic resin^3,4^. Also, flowable composites have reduced viscosity which increases the resin loading in the composite, affecting its mechanical/physical performance. Hence, to increase the performance of dental composites, focus in the past has been on variations in the filler material, size, shape, loading and enhancing filler-fibre interfacial bond^4^. Addition of short glass fibres in dental composites has been investigated over 40+ years^5–7^, wherein the mechanical properties are enhanced to a great extent over the past 15+ years^8,9^. The efficiency of fibre reinforced dental composites is dependent on fibre length, the bond strength between fibre and resin, fibre aspect ratio (AR), the orientation of fibres to the load path and quantity of fibres used^10^.

Light cured resin-based composites generally suffer from dimensional instability which could cause marginal gaps leading to leakage, secondary caries and loss of mechanical properties^11^. Dimensional stability during initial setting depends on the extent of polymerisation which in turn is dependent on several intrinsic factors (type and amount of monomer, photo-initiator, co-initiator and fillers), curing kinetics (curing intensity, light tip size, power density and curing lamp profile) and extrinsic factors (irradiation time, irradiation distance and light guide tip positioning)^12^. Dimensional stability could also be affected during the life of the restoration due to its presence in an aqueous acidic oral environment subjected to thermal and mechanical cycling^13,14^. The dimensional stability involves evaluation of the depth of cure, volumetric shrinkage, polymerisation stress, exotherm, water sorption, solubility, and thermal expansion coefficient.

For posterior restoration, the primary drawback of light-cured composites is its inability of curing light to penetrate to a greater depth, which leads to time-consuming traditional 2 mm incremental layering. Commercially available bulk-fill flowable composites can cure up to 4 mm. Volumetric shrinkage is an inherent property of resin-based dental composites wherein the inter-atomic distance between the molecules is reduced during polymerisation and a nano-scale free volume is created, leading to overall material shrinkage^15,16^ in the range of 1.5–6.0%^17^. This shrinkage exerts internal contraction within the material leading to residual stress, which tends to dislocate the restoration from the cavity walls creating marginal gaps. Most of the shrinkage occurs during the pre-gel stage where the material is still able to flow (viscous stage), which negates the induced stresses. However, during the post-gel phase, the material is almost solidified (viscoelastic stage) gaining elastic properties and further material curing (solidification) results in the generation of residual stress^18^. The process of polymerisation is exothermic and excessive heat conducted through the remaining dentine during a restoration could result in irreversible damage to the pulp if the temperature is greater than 5.5 °C at the pulp^19^. Exotherm could be generated through the inherent material property (polymerisation) or exotherm of the light-curing unit or clinician (polishing with high-speed units in the absence of coolant). Higher resin volume fraction (flowable composites) and irradiation above 1000 mW/cm^2^ could result in a higher degree of conversion but suffer from greater volumetric shrinkage, shrinkage stress and higher exotherm^19^. Thus, polymerisation shrinkage is defined as a factor of shrinkage, material modulus and curing kinetics.

The excessive difference in relative hygro-thermal expansion between restorative composite and dentine could result in debonding and microleakage, leading to premature restoration failure. Also, moisture/water absorption through hydrolysis weakens the resin-filler interface by breaking the hydrolytic bond between them, thus reducing the restoration mechanical and physical properties^20,21^. ISO 4049^22^ recommends the water sorption and solubility to be less than 40 µg/mm^3^ and 7.5 µg/mm^3^ respectively, for the dental composite restorative material. Combined with fillers, a higher glass fibre content (10–25% volume) was able to reduce the water sorption considerably^23,24^. However, in another study, compositions with a low volume fraction of fibres (4–6%) had a negligible effect on the water sorption^25^. Similar to the hygroscopic effect, a large difference in thermal expansion coefficients (CTE) between tooth and restoration causes debonding, microleakage and premature failure^26,27^. CTE of enamel and dentine are 17 × 10^–6^/ºC and 11 × 10^–6^/°C^28^, dental neat resin—110–190 × 10^–6^/°C, glass particles—0.50–6.0 × 10^–6^/°C and dental composites—20–80 × 10^–6^/°C^29^. Generally, the oral environment temperature range is between 0 and 67 °C and CTE is inversely proportional to filler loading. Effect of thermal changes due to different CTEs is critical for bond durability and needs to be investigated.

Several studies have been conducted in the past on physical characterisation of commercial and experimental filler/fibre-reinforced dental composites^30–33^. However, the effect of replacing a small number of glass fillers with glass fibres with the same resin system on the physical properties has not been attempted. Recently, based on optimisation of fibre length and weight fraction through mechanical characterisation, new experimental flowable short S-Glass fibre reinforced dental composites were introduced^9^. Therefore, this study is aimed at investigating the effects of replacing 5 wt% fillers with 5 wt% short S-Glass fibres with two aspect ratios on the dimensional stability of flowable dental composites.

## Materials and methods

The flowable dental composite system consisted of 80 wt% UDMA-20 wt% TEGDMA (Esstech, Inc., USA) based resin reinforced with 0.7 µm strontium glass particles (SDI Limited, Australia) and S2-Glass fibres from AGY, USA were cut (Engineered Fibres Technology, LLC, USA) to lengths of 250 µm and 350 µm with aspect ratios (AR) of 50 and 70. S2-Glass fibre surfaces were etched (4 h in 37% HCl) and silane treated (1 h in 2% TMSPMA solution) before being blended with the resin system as described by Cho et al.^34^ which enhanced the mechanical and chemical interlocking of resin with glass fibres. The detailed resin composition corresponding weight fractions for this study are listed in Table 1.

**Table 1:** Resin composition.

Material	Wt%
UDMA (urethane dimethacrylate)	79.4
TEGDMA (triethylene glycol dimethacrylate)	19.5
Camphorquinone—photoinitiator	0.2
EDB (ethyl 4-dimethylaminobenzoate)—co-initiator	0.5
BHT (bisphenol-A-glycidyl dimethacrylate)—inhibitor	0.5

FlackTek Speedmixer (DAC150FV, FlackTek Inc, USA) was used to mix the resin and fillers under vacuum twice at 3500 rpm for 20 s and the three compositions formulated are detailed in Table 2. Samples from all the three groups were light-cured 1500 mW/cm^2^ using Radii plus (SDI, Australia) for 65 s.

**Table 2:** Three groups of experimental composition.

Group	Resin wt%	Aerosil R202 wt%	Strontium filler (0.7 µm) wt%	S-2 Glass fibre (Ø 5 µm)		
				Wt%	Length (µm)	Aspect ratio
Gr-A	43.4	1.6	55	NA	NA	NA
Gr-B	43.4	1.6	50	5	250	50
Gr-C	43.4	1.6	50	5	350	70

Samples of 15 mm × 4 mm × 3 mm (n = 5) were manufactured in a split aluminium mould between two transparent films and the dental composite was injected into the mould sandwiched between two glass slides. Injected material was light-cured from one end and the specimen was separated from the mould. Uncured material from the other end was scraped off using a plastic spatula and samples were placed in distilled water and transferred into a dark incubator at 37 °C for 24 h. The samples were polished (Struers Labopol-5) using 1200 grit paper and tested on Struers DuraScan-80 as per ISO 10477^35^, with a load of 500 g and a dwell time of 5 s and Vickers hardness number (VHN) was recorded. Depth of cure profile (0.5 mm increment) was created over the cross-section of each sample by generating sequential hardness sampling and the data was compared with the hardness at the curing surface. Depth of cure was calculated at a value when the hardness reduced below 80% of the maximum hardness value (at curing surface).

Archimedes method (buoyant force principal) was used to determine volumetric shrinkage as per ISO 17304^36^ wherein the material was weighed (analytical balance GH-252, AANDD, Japan) in air and water before and after polymerisation. The weights of uncured paste (n = 12) and cured samples (n = 6) were measured using density determination kit (Radwag, USA) in both air and buoyancy medium and subsequent densities were computed using Eq. (1),

1 $$ \rho = \frac{{m_{air} * \rho_{buoyancy} }}{{m_{air} - m_{buyoancy} }} $$

where ${m}_{air}$ and ${m}_{buyoancy}$ are the mass of samples in air and buoyancy medium respectively, and ${\rho }_{buoyancy}$ is the density of the buoyancy medium. Volumetric shrinkage ‘S’ in percentage terms was calculated using Eq. (2),

2 $$S=\left(\frac{\stackrel{-}{{\rho }_{c}}-\stackrel{-}{{\rho }_{u}}}{\stackrel{-}{{\rho }_{c}}}\right)*100$$

where $\stackrel{-}{{\rho }_{c}} and \stackrel{-}{{\rho }_{u}}$ are the mean densities of polymerised sample and unpolymerized paste.

**Figure 1:**
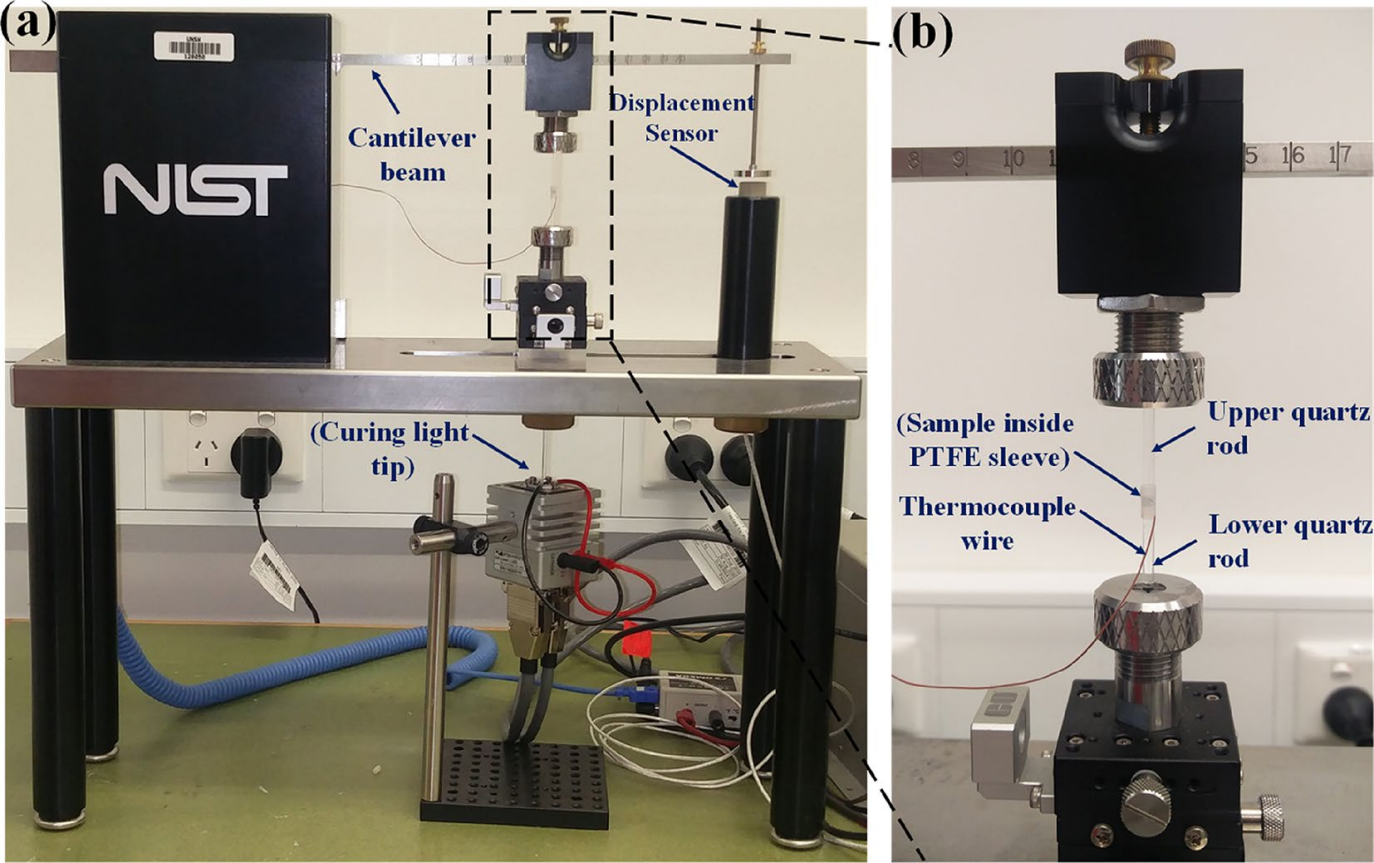
(**a**) Cantilever beam based NIST instrumentation for polymerisation stress and thermocouple based exotherm measurement system (**b**) Close-up view of sample mounting zone showing the sample embedded between two quartz rods and thermocouple wires.

Evaluation of polymerisation stress and exotherm was carried out using a cantilever beam tensometer (NIST, USA)^37^ designed specifically for light-cured materials as shown in Fig. 1a. Teflon sleeve of 2.5 mm diameter was mounted between two quartz rods with a spacing of 2 mm (sample height). The top rod was clamped to the cantilever beam of the tensometer while the bottom rod was clamped to the rigid base. An injection hole and a small air-vent hole were drilled in the Teflon sleeve before mounting, to enable material insertion through a syringe. A single blue LED (460 nm, Arroyo 226, Arroyo Instruments, USA) was installed on a mount, controlled by a controller (Laser diode controller 6340, Arroyo Instruments, USA) and was used to irradiate the sample through the bottom quartz rod. A simultaneous exotherm was measured using a T-type thermocouple (0.08 mm diameter, TC Measurement & Control Pty Ltd., Australia), which was inserted at the centre of the sample as shown in Fig. 1b. Labview (National Instruments, USA) based software was used as an interface to acquire the synchronised polymerisation stress and exotherm data for 900 s at a sampling rate of 1 Hz. Once the sample was irradiated, the shrinkage in the material caused the cantilever beam to deflect down, and this deflection was recorded at the end of a cantilever beam with a displacement sensor and polymerisation stress was evaluated based on the beam formula^38^. Samples within the groups were selected in random and observed under SEM to verify uniform distribution and random orientation of fibres.

Water sorption and solubility tests were conducted as per ISO 4049^22^ wherein samples from three groups were of 15 mm diameter and 1 mm thick (n = 5) each and were irradiated on each side with 8 overlapping exposures. After curing, the sample was removed from the mould, edges lightly cleaned with 1200 grit sandpaper, and dimensions were recorded. Samples were stored (dry) in the dark incubator at 37 °C until a stable constant mass (m_1_) was obtained (analytical balance GH-252, AANDD, Japan). Samples were then immersed in distilled water on a rack with 4 mm separation between them at 37 °C for 7 days and weight recorded (m_2_). Samples were again stored on the rack (dry) for 4 weeks in a dark incubator at 37 ºC until a stabilised weight was reached (m_3_). Water sorption (µg/mm^3^) and solubility (µg/mm^3^) were computed, as shown in Eqs. (3) and (4), where V is the volume of the material,

3 $$ W_{sp} = \frac{{m_{2} - m_{3} }}{V} $$

4 $$ W_{sl} = \frac{{m_{1} - m_{3} }}{V} $$

Linear thermal properties were evaluated using horizontal push-rod dilatometer (NETZSCH 402C) at temperatures from 25 to 80 °C in an inert atmosphere (argon at 50 ml/min) with an increment of 5°/min as per ASTM E228-17^39^. Calibration using Al_2_O_3_ (baseline) was conducted and the cylindrical dental composite samples of length 12 mm and 4.5 mm diameter (n = 5) were used while data was recorded at 4 Hz. Once the furnace reached the required peak temperature (first heating cycle), the system was naturally cooled to room temperature. A second consecutive heating cycle was applied without removing the sample from the furnace with the same set of system parameters and the thermal expansion coefficients were recorded.

Mean values of depth of cure, polymerisation stress, exotherm, water sorption, solubility and linear thermal expansion coefficients were statistically analysed with one-way ANOVA followed Tukey’s post hoc test (p = 0.05) using Origin Pro 8.6. Pearson correlation (r) with a 2-tailed test of significance between the 5 wt% particle size various physical parameters were also determined.

## Results

**Figure 2:**
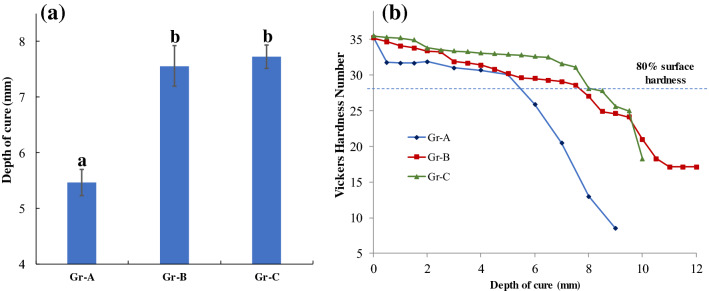
(**a**) Mean and standard deviation of the depth of cure. Groups with the same alphabets are not significantly different from each other (p > 0.05); (**b**) variation of Vickers hardness number (VHN) along the depth of the sample from the curing surface. Horizontal dotted line represents 80% of surface microhardness.

The mean values of curing depth with for the tested groups with standard deviations are presented in Fig. 2a. In comparison with Gr-A, Gr-B and Gr-C had a 38% and 41% increase in depth of cure from 5.47 mm to 7.55 and 7.72 mm, respectively. There was a statistically significant difference between the means of the groups, determined by one-way ANOVA (p < 0.01). Tukey’s post hoc test revealed that there was a statistically significant difference between Gr-A and Gr-B as well as Gr-A and Gr-C. Though a small difference of 0.17 mm depth of cure was observed between groups Gr-B and Gr-C, no statistically significant difference (p = 0.74) was observed. Figure 2b shows the variation of VHN over the curing depth. VHN at the curing surface was around 35 for all the three groups. However, at a depth of 0.5 mm, VHN of Gr-A samples was reduced by 10% and a steep decline in the hardness was observed from 5 mm. Groups B and C had a similar slope until 8 mm and then hardness reduced drastically until the end of the sample.

**Figure 3:**
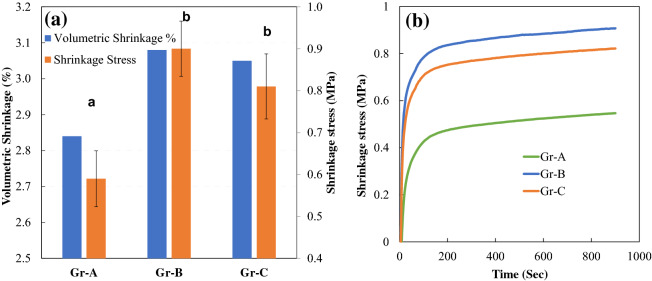
(**a**) Mean polymerisation stress for three groups. Groups with the same alphabets are not significantly different from each other (p > 0.05) (**b**) Variation of polymerisation stress over time for 900 s.

Volumetric shrinkage and polymerisation stress of the three groups tested are shown in Fig. 3a. In comparison with Gr-A, volumetric shrinkage increased by 8.3% and 7.3% for groups B and C, respectively while polymerisation stress increased by 52.7% to Gr-B and 37.6% to Gr-C compared to Gr-A as shown in Fig. 3a. A statistically significant difference between means of groups was observed (p < 0.01) through ANOVA. Tukey’s post hoc test suggested that the means differences were statistically significant between Gr-A and Gr-B (p < 0.01), Gr-A and Gr-C (p < 0.01) and Gr-B and Gr-C (p < 0.05). Polymerisation stress as a function of time for three groups is shown in Fig. 3b, where sharp curves were observed for groups with fibres and a lower rate of increase for Gr-A. The overall magnitude of the polymerisation stresses was between 0.6 and 0.9 MPa. For all the three groups, it is seen that polymerisation stress increases rapidly till the curing light was turned off at 65 s and gradually increases till 900 s.

**Figure 4:**
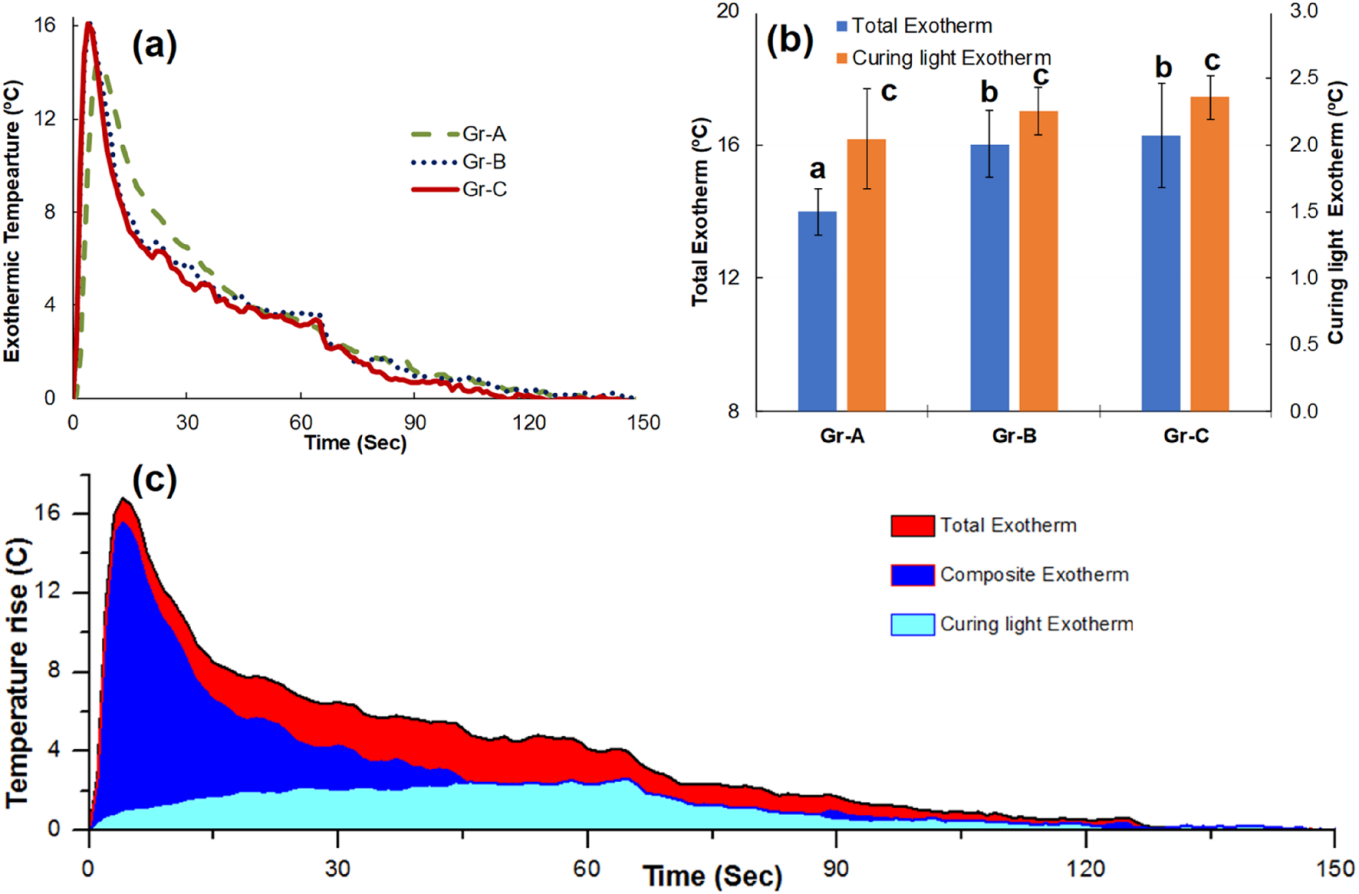
Exotherm profile for (**a**) Variation of total exotherm over time for first 150 s (**b**) Mean and standard deviation values of first and second irradiation exotherm for three groups where the groups with the same alphabets are not significantly different from each other (p > 0.05) (**c**) Superposition of temperature–time profile for total exotherm, composite exotherm and curing light contribution for the sample from Gr-C.

Variation of exotherm over time for the three groups is shown in Fig. 4a where a rapid increase in temperature is seen at the beginning of irradiation and gradually reduced to near 0 °C at 150 s and continued the same till the end of the test. Gr-A reached its peak temperature at 6 s and Gr-B and Gr-C reached their peak temperatures at 4 s. A consecutive second illumination was applied to measure the exotherm of the curing light alone in the presence of cured restorative material, as presented in Fig. 4b. It is seen that Gr-B and Gr-C had 14.5% and 20.2% higher exotherm than Gr-A. Curing light exotherm was measured during the second consecutive irradiation and found to be around 2 °C for all groups as shown in Fig. 4b. Figure 4c shows the superposition of total exotherm, composite exotherm and curing light temperature contribution. It is seen that the composite exotherm reduces to a minimum value at around 45 s suggesting completion of polymerisation of the composite. The population means of all the three groups were significantly different (p < 0.01) as per ANOVA and Tukey post hoc suggested that the means differences between the groups were significantly different (p < 0.01) for Gr-A & Gr-B and Gr-A & Gr-C. However, the means difference was not significant for Gr-B and Gr-C (p = 0.32).

Water sorption, solubility, and their associated weight difference in the percentage of the tested groups are presented in Table 3. Water sorption was around 19 µg/mm^3^ for all the three groups with a mean weight increase of around 1.2%. Solubility was around 0.6% with a mean weight decrease of 0.35%. The population means were not significantly different for water sorption (p = 0.81) and solubility (p = 0.59). Tukey post hoc also suggested that the means difference was not significant (p > 0.05) between the groups for both water sorption and solubility.

**Table 3:** Water sorption and solubility of tested groups.

Group	Water sorption (µg/mm^3^)	Water sorption weight increase %	Solubility (µg/mm^3^)	Solubility weight decrease %
Gr-A	19.17 ± 0.73	1.78 ± 0.04	0.43 ± 0.30	0.27 ± 0.02
Gr-B	18.96 ± 0.66	1.13 ± 0.03	0.64 ± 0.30	0.39 ± 0.02
Gr-C	19.21 ± 0.52	1.13 ± 0.05	0.60 ± 0.39	0.37 ± 0.02

**Figure 5:**
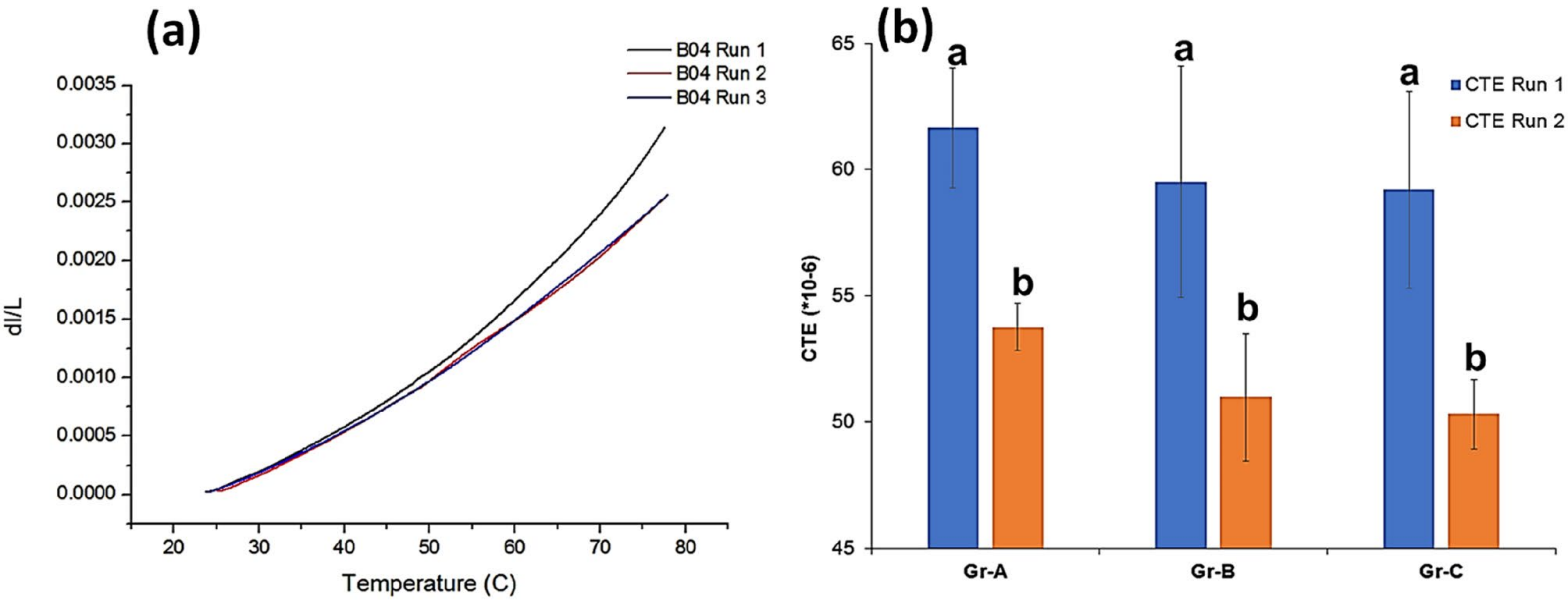
(**a**) variation of thermal strain between 25 and 80 °C intervals of sample B04 (run 1) and subsequent two runs (runs 2 and 3) (**b**) Mean and standard deviation of CTE for three groups between 25 and 80 °C at 5 °C intervals from run 2. Groups with the same alphabets are not significantly different from each other (p > 0.05).

Variation of CTE (Thermal strain vs. temperature) between the first and subsequent heating cycles is shown in Fig. 5a for a representative sample B04, from Gr-B. It was observed that the thermal strain (dl/L) was higher at 3.14E−3 for the first heating cycle and 2.54E−3 for both second and third heating cycles, respectively. Thermal strain vs. temperature curves shows a distinct variation between the first and subsequent two heating cycles. Figure 5b shows the difference of CTE between the first and second heating cycles for the three groups. It is seen that the mean CTE value for groups B and C reduced by 5.2% and 6.4% respectively. ANOVA revealed that the statistical means were not significantly different (p > 0.05) between the three groups.

## Discussion

Large dimensional variation of the restorative composite due to external/internal/curing kinetics conditions could lead to debonding of the restoration, microleakage and restoration failure. Several studies^1,9,10,31,33,40,41^ have analysed the effect of a small volume of short fibres on mechanical and physical properties along with particulate fillers or addition of fibres to commercial composites. The effectiveness of surface etching, random orientation and uniform distribution were also evaluated where the interfacial shear strength measured by microdroplet pull-out study was enhanced by 39.6%^34,42^. With the replacement of 5 wt% glass particles with glass fibres (50AR and 70AR), the flexure strength and flexure modulus increased 22.3% and 9.7% respectively, compared to the filler only group^9^. The current study focused on the physical properties characterisation of these three groups based on the glass fibre surface treatment by Cho et al.^34^.

The ability of the curing light passing through the resin composite decreases over the thickness as the light gets scattered between the organic resin and inorganic reinforcement filler particles due to variation of refraction indices between them^43^. A higher weight fraction of glass filler particles would result in higher light scatter causing a decrease in total energy received and thus causing insufficient polymerisation of the monomer. In this study, the individual particle volumes of filler, 50AR and 70AR fibres were 0.14, 4909.00 and 6872.00 mm^3^, respectively. Hence, the number of particles was reduced by replacing 5 wt% fillers with 5 wt% long fibres and additionally the glass fibres transmit light through its length in different directions when randomly oriented, causing higher light penetration through the thickness, resulting in a degree of conversion, and curing depth.

All the three groups exceeded the minimum clinical requirement of 2 mm depth of cure for dental composites. Microhardness values reduced incrementally compared to the irradiation surface, which had the highest microhardness. This test was aimed at understanding the relative variation between the tested groups, rather than arriving at absolute values. Commercial manufacturers do not recommend more than 4 mm increment filling to minimise the intensity of polymerisation stress. Glass fibres tend to absorb and scatter the light efficiently and previous studies have also shown increased curing depth^31,41^ of compositions with glass fibres. In the clinical scenario, the curing light may not be accurately placed over the restorative surface which could affect the curing depth. However, the addition of glass fibres could partially compensate for the inaccurate light placement of light through enhanced light distribution within the material. Other resin properties such as shade, scattering of light and coefficient of absorption need to be considered^31^.

Shrinkage during polymerisation in viscoelastic dental composites occurs pre-gel and post-gel phases. In the pre-gel phase, the material is viscous and deformable, thereby alleviating any stress generation. However, in the post-gel phase, the material begins to solidify and is not-deformable, gaining higher elastic properties^37,44^. Total volumetric shrinkage measurement encompasses both pre-gel and post-gel phase and is generally recommended which provides a holistic approach. During irradiation, the polymerisation begins from the curing surface towards the depth and any shrinkage (volumetric) below the surface exerts internal stresses at the walls which could create debonding of the restoration. Shrinkage volume and polymerisation stress are some of the primary factors for marginal leakage and secondary caries^17^. Flowable composites with higher resin content relatively have high volumetric shrinkage and low shrinkage stresses. It is known from many studies that the elastic modulus increases with the addition or replacement of a small volume of fibres^1,9,45,46^. It has also been established that volumetric shrinkage is directly proportional to the elastic modulus of the material^44,47^ where the increase in modulus is attributed towards the large particle size of fibres which improves the load-carrying capacity and toughness of the structure. Behl et al.^9^ in their study had reported that the flexure moduli for the three groups tested in this study were 5.29 GPa, 6.47 GPa and 6.17 GPa. A similar trend was observed in the current study where volumetric shrinkage varied in proportional to the flexure modulus^9^ which increased for groups B and C. Both the volumetric shrinkage and flexure modulus were high for Gr-B and Gr-C; however, no statistically significant difference was observed in the groups between 50AR (Gr-B) and 70AR (Gr-C) fibres (p < 0.05).

Many factors influence volumetric shrinkage and polymerisation stress such as resin type, resin quantity, type of filler, filler size, filler quantity, irradiation time, curing light profile, curing time, C-factor (geometry and size of the cavity) and the test set-up^38,44^. It is recommended that shrinkage stress values need to be compared only with a similar test setup as the system parameters could highly influence the results. As seen in Fig. 3b, all groups experienced rapid shrinkage stress development during the first 65 s where the stresses were significantly higher for groups B and C and are in agreement with earlier studies^33,40^. Past studies have also shown a direct correlation of shrinkage volume and shrinkage stress^48,49^ and similar relationship is observed in the current study. It is known that shrinkage stress and shrinkage volume are directionally proportional to the material elastic modulus^50,51^, which is observed in the current study. The flexure moduli for the three groups are: 5.29, 6.47 and 6.17 GPa^9^, measured shrinkage volume is 2.84%, 3.08% and 3.05% and shrinkage stress of 0.59 MPa, 0.9 MPa and 0.81 MPa respectively, which correlates with the earlier studies \^50,51^.

Temperature due to exotherm increased during the first 4–6 s and returned to baseline temperature after 140 s. Temperature increase during polymerisation is a combination of exotherm from restorative composite and the curing light. To separate these two effects, consecutive second irradiation was applied to the material immediately after the polymerisation (after returning to baseline temperature), where the temperature rise due to curing light is measured which was 2 ºC for all the groups. Temperature rise and the rate of reaction during photo-polymerisation is directly proportional to the amount of resin volume, curing light and the extent of degree of conversion^52,53^. A past study^54^ has established that the time required to reach the maximum temperature in the polymerisation reaction process could be approximated as the vitrification point. Vitrification is the point where the material acquires elastic properties and a clear transition from the pre-gel to post-gel phase^55^. Gr-A samples, in the absence of fibres, had a peak temperature at 4 s in comparison with 6 s for Gr-B and Gr-C. This behaviour demonstrates the enhanced gelation and corresponding vitrification has been advanced by 2 s, demonstrating the enhanced energy dispersion and increase of curing rate with the addition of fibres. However, early vitrification also comes with the challenge of higher polymerisation stress^56^, which is evident in the current study. No significant difference in exotherm variation was observed between Gr-B and Gr-C (p < 0.05). Flowable composites have higher resin content and additionally, UDMA and TEGDMA enables a higher rate of polymerisation^57^, hence higher value of exotherm, up to 16 °C was observed in all the three groups.

In this study, as the resin volume and curing light intensities are kept constant, the marginal increase in exotherm (16 °C) and early peak-temperature time (4 s) for Gr-B and Gr-C could be attributed towards a higher degree of conversion due to high energy dispersion during polymerisation due to reduced particle count, which is comparable with the earlier work^53^. One advantage of higher exotherm is that it would result in greater thermal expansion and an increase in free volume, which helps in alleviating the residual polymerisation stresses. Hence, if the remaining dentine in the tooth to be restored is greater than 1.0 mm, the higher exotherm would help ensure the marginal integrity of the restoration^58,59^.

For all the three groups, the resin volume fraction and total filler volume were kept constant and the thickness of the samples was just 1 mm as per ISO 4049. Due to small specimen thickness and 16 irradiations for each sample, degree of conversion could be considered same for all three groups, hence the minimum variation of water absorption and solubility is observed between the tested groups. The soluble materials in the dental composite are the uncured free monomers, photo-initiators and plasticisers/accelerators^60^. In this study, 20 wt% TEGDMA, a low viscosity diluent having a low molecular weight (286 g/mol) was mixed with a highly viscous UDMA (80 wt%) with a high molecular weight (470 g/mol). Major factors influencing water sorption are storage temperature, immersion duration, degree of conversion, the three-dimensional molecular structure of monomers, amount of residual monomer molecules in the polymerised composite, free space (shrinkage) available within the 3D structure after polymerisation, hydrophilic nature of the monomer and solubility index of monomer system^57,61^. Also, the hydrophilic groups present in the monomers such as OH (UDMA), NH (UDMA) and ethylene groups (TEGDMA) contribute to water sorption. Hence water sorption and solubility values for the three groups in the current study were similar and also comparable with the earlier studies on the base monomers^57^. Solubility is dependent on the quantity of water ingress, the amount of leachable molecules and pendant double bonds and compared to UDMA, TEGDMA is prone to higher solubility^57^. Hence, due to the presence of TEGDMA, even though with a higher degree of conversion, slightly higher solubility was observed in this study. As water sorption and solubility are resin dominated properties, both particle size and fibre aspect ratio have a limited effect. Water sorption and solubility of all the three groups were under the threshold of 40 µg/mm^3^ and 7.5 µg/mm^3^ respectively as per ISO 4049^22^.

Photocured dental composites always possess a certain degree of volumetric shrinkage, which results in residual stress. This residual polymerisation stress tends to debond the restoration from the dentine walls, which could result in restoration failure and microleakage. However, water sorption contributes to hygroscopic expansion which could partially alleviate the residual polymerisation stresses and increase the sealing between dentine and restoration^60^.

Thermal expansion is the molecular movement of the constituents in a mixture due to variation in their thermal energies. Thermal induced stresses due to mismatch in the CTEs of constituent materials could result in debonding of the restoration and microleakage. While measuring CTE, the value obtained in the first heating cycle is generally not reproducible and reliable and is recommended that CTE be measured on the cooling cycle or during the second heating cycle^26,62,63^. During the first cycle (run), additional polymerisation of the matrix, settling, stress relief within the material or alteration of the specimen morphology occurs^26,64^. The CTE of organic monomers ranges between 110 and 190 × 10^–6^/°C^32,65^ and between 0.16 and 0.73 × 10^–6^/°C for inorganic glass fillers^66^ where S-Glass has a CTE of 0.16 × 10^–6^/°C. The lower CTE for Gr-B and Gr-C is attributed towards (i) a higher degree of conversion^67^ and (ii) randomly oriented rigid glass fibres preventing the free monomer movement, similar to an earlier study^27^. A higher degree of conversion results in an increased number of carbon–carbon covalent bonds between the polymer chains and this increased cross-linking reduces the polymer chain movement at higher temperatures. Hence, a 5.2% and 6.4% decrease in CTE was observed for groups Gr-B and Gr-C.

Correlation analyses were used to examine the relationship between the particle size and different physical parameters of short S-Glass reinforced flowable dental composites. A strong positive correlation was observed for the depth of cure (0.968), volumetric shrinkage (r = 0.923), polymerisation stress (r = 0.846), exotherm (0.999), and CTE (0.961) which demonstrates the strong influence of replacement of fillers with fibres on the physical properties.

## Conclusions

Adequate physical and mechanical properties at the depth of a thick restoration are required for long life and to withstand combined hygro-thermal-impact loadings in-vivo in the complex acidic environment of the oral structure. Promising increase in the mechanical properties due to addition has led to the manufacture of photocured packable fibre reinforced dental composites over the past decade. Usage of short glass fibres in flowable bulk filled composites has enabled a reduction in incremental steps and increased ease of placement while maintaining enhanced mechanical properties. Modification of constituents (shape, size, material and quantity) in a composite has complex material behaviour where the properties might increase/decrease or stay constant. In the current study, with the replacement of 5 wt% fillers with small aspect ratio, depth of cure increased due to higher degree of polymerisation due to enhanced light penetration in the material measured by micro-hardness. Because of this, a smaller number of residual monomers in the material led to a lower thermal expansion coefficient which is beneficial. However, fibre replacement had a detrimental effect on the volumetric shrinkage, exotherm and shrinkage stress. Those can be attributed to a higher degree of conversion by increased particle size. Higher shrinkage stress is attributed to the higher elastic properties of the material. Also, fibre replacement had had a negligible effect on water sorption and solubility. The effect of fibre length on the physical properties were negligible.
